# In Memoriam: Carl-Albrecht Haensch, M.D.

**DOI:** 10.1007/s10286-024-01017-4

**Published:** 2024-02-26

**Authors:** Jens Jordan, Andrea Meier, Christina Haubrich, Rolf R. Diehl, Max J. Hilz

**Affiliations:** 1 Institute of Aerospace Medicine, German Aerospace Center (DLR), Linder Hoehe, 51147 Cologne, Germany; 2 Medical Faculty, University of Cologne, Cologne, Germany; 3 Department of Neurology, Medical Faculty, RWTH Aachen University, Aachen, Germany; 4 NeuroPraxis Düsseldorf, Düsseldorf, Germany; 5 Medical Faculty, RWTH Aachen University, Aachen, Germany; 6 Department of Neurology, Alfried Krupp Hospital, Essen, Germany; 7 Department of Neurology, University of Erlangen-Nuremberg, Erlangen, Germany; 8 Icahn School of Medicine at Mount Sinai, New York, NY USA

Carl-Albrecht Haensch, a compassionate physician, seasoned clinician scientist, and dear colleague and friend left us on November 18th 2022 at the age of only 58 years. He was head of the clinical neurology department at Mariahilf Kliniken in Moenchengladbach and adjunct (apl.) professor of neurology at the University of Witten/Herdecke in Germany. Throughout his career, mechanisms and treatment of autonomic nervous system disorders and interactions between sleep and autonomic control mechanisms in human beings were important topics of his work. He also devoted much time and effort to teaching autonomic nervous system physiology and clinical management of autonomic nervous system disorders to students and physicians in training. Moreover, over many years, he had an important role in the German Autonomic Society (Arbeitsgemeinschaft Autonomes Nervensystem) where he had served in various leadership positions. In his private life, Carl-Albrecht Haensch enjoyed attending rock concerts and major league soccer games of the local team VFL Borussia Mönchengladbach with his wife and two sons. Owing to his curiosity and broad interests, he and his family explored other cultures during trips to the Americas and Asia. He was a passionate hobby chef with a particular love for French cuisine (Fig. 1).

**Fig. 1:**
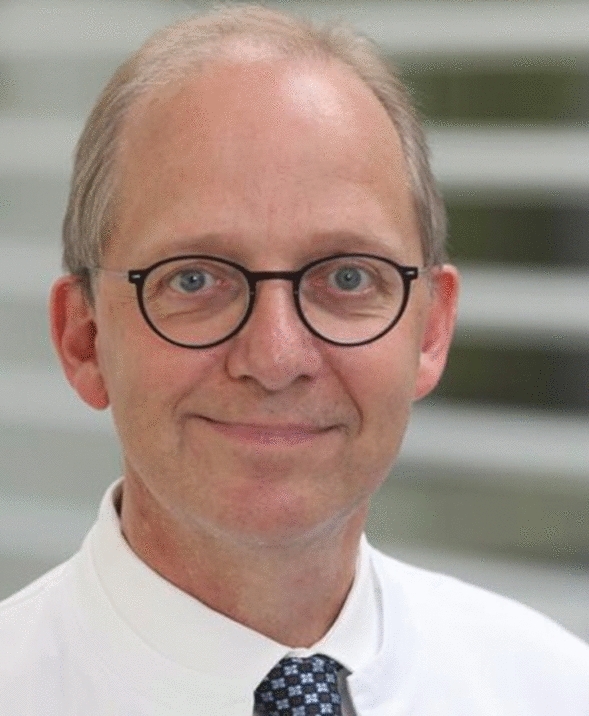
Carl-Albrecht Haensch, M.D. (Copyright Detlef Ilgner)

Carl-Albrecht Haensch graduated from medical school at the Heinrich Heine University in Duesseldorf, Germany, and completed a thesis on mechanisms of diabetic neuropathy at the German Diabetes Center. He then trained in clinical neurology and neurophysiology, sleep medicine, and pain medicine in Wuppertal under the supervision of Professor Johannes Joerg. He acquired the formal qualification to serve as professor (habilitation) from the Medical Faculty of the University Witten/Herdecke, which he later joined as adjunct professor. In 2010, Carl-Albrecht Haensch received the prestigious Robert Wartenberg Prize from the German Society of Neurology (Deutsche Gesellschaft fuer Neurologie) for his work on neuropathic changes in patients with the postural tachycardia syndrome (POTS).

Carl-Albrecht Haensch’s autonomic nervous system research centered on clinically relevant topics. He was a particular expert in the pathogenesis and treatment of POTS. Using cardiac I-123-metaiodobenzyl-guanidine (MIBG) imaging and skin biopsies, he and his coworkers provided evidence for neuropathic changes in some patients with POTS [1–3]. Better mechanistic understanding, such as that gained by Carl-Albrecht Haensch, may beget more targeted therapies for patients with POTS in the future. He also observed changes in sleep quality and sleepiness in patients with POTS, which could have an important bearing on their quality of life [4].

However, Carl-Albrecht Haensch also studied autonomic involvement in other disorders. For example, in a patient diagnosed with chronic complex regional pain syndrome type I of the left hand, he observed a substantially reduced MIBG uptake in the affected limb compared to the contralateral arm [5]. The study provides insight into the role of the sympathetic nervous system in the pathogenesis of chronic complex regional pain syndrome type I and could have diagnostic and therapeutic implications. Other studies assessed autonomic abnormalities in patients with multiple sclerosis [6] and Parkinson’s disease [7]. Carl-Albrecht Haensch’s astute clinical observations were crucial in discovering a new genetic cause of ganglionic acetylcholine receptor alpha-3 subunit deficiency, which appeared in print few months before his death [8].

Autonomic nervous system and medications interact in a bidirectional fashion. While influences of various clinical conditions such as renal or hepatic dysfunction on pharmacokinetics are routinely tested during clinical drug development, neurological conditions, particularly neuropathies affecting the autonomic nervous system, are somewhat neglected. Carl-Albrecht Haensch and coworkers contributed important research addressing the interaction between pharmacotherapy and the autonomic nervous system. One study showed that transdermal estrogen uptake did not differ between patients with neuropathies and control persons [9]. Another report suggested that treatment with bortezomib could be a rare cause of severe autonomic failure [10].

Carl-Albrecht Haensch made sure that research findings do not get stuck in academia and are widely applied in the trenches of clinical medicine. He edited a widely distributed book on autonomic medicine in German language, which appeared in its second edition in 2022 [11]. He also contributed to clinical guidelines that are highly relevant to patients with disorders affecting the autonomic nervous system, such as a clinical guideline on the management of erectile dysfunction [12].

Carl-Albrecht Haensch will be remembered as a patient-oriented neurologist, clinical researcher, and teacher with profound insights into many aspects of neurology, including sleep disorders, pain, or stroke, to mention just a few of his areas of interest. He was a master in intertwining these topics with his passion for the autonomic nervous system. His colleagues, students, and many scientists and friends will always have a good memory of Carl-Albrecht Haensch.

## Data Availability

Not applicable.
